# Revisiting the Postulates of Etiological Models of Eating Disorders: Questioning Body Checking as a Longer-Term Maintaining Factor

**DOI:** 10.3389/fpsyt.2021.795189

**Published:** 2022-01-13

**Authors:** Vanessa Opladen, Maj-Britt Vivell, Silja Vocks, Andrea S. Hartmann

**Affiliations:** ^1^Department of Clinical Psychology and Psychotherapy, Institute of Psychology, Osnabrück University, Osnabrück, Germany; ^2^Department of Psychology, Experimental Clinical Psychology, University of Konstanz, Konstanz, Germany

**Keywords:** body checking, emotion, shape and weight concerns, theory of eating disorders, body image

## Abstract

Body checking (BC) is not only inherent to the maintenance of eating disorders but is also widespread among healthy females. According to etiological models, while BC serves as an affect-regulating behavior in the short term, in the longer term it is assumed to be disorder-maintaining and also produces more negative affect. The present study therefore aimed to empirically examine the proposed longer-term consequences of increased BC. In an online study, *N* = 167 women tracked their daily amount of BC over a total of 7 days: Following a 1-day baseline assessment of typical BC, participants were asked to check their bodies in an typical manner for 3 days and with a 3-fold increased frequency for 3-days. Before and after each BC episode, the impact of BC on affect, eating disorder symptoms, general pathology and endorsement of different functions of BC was assessed. Participants showed longer-term consequences of increased BC in terms of increased negative affect and general pathology, while eating disorder symptoms remained unaffected. In the case of typical BC, participants showed decreased general pathology and anxiety. Furthermore, the endorsement of a higher number of BC functions led to increased negative affect and an increased amount of typical BC. The findings support the theoretically assumed role of maladaptive BC in maintaining negative emotion in the longer term. However, though requiring replication, our finding of positive effects of typical BC calls into question the overall dysfunctionality of BC among non-clinical women who are not at risk of developing an eating disorder.

## Introduction

*Body checking* (BC) describes the repeated evaluation of one's own body (1). It occurs in several mental disorders such as body dysmorphic disorder [BDD (2)] or illness anxiety disorder (3), but is most commonly known in the context of eating disorders (EDs). Patients with EDs engage in BC to obtain information about their size, shape, and weight (4, 5). Etiological models of EDs assume a high relevance of BC, and empirical research has demonstrated that BC is a contributing factor to the development (6) and maintenance (7) of ED symptoms. In the context of ED pathology, BC includes multiple strategies of monitoring one's general appearance in an idiosyncratic manner (8). Common strategies include repeated weighing or mirror gazing, measuring or pinching body parts, or trying the fit of clothes to assess one's body size (9). It may also involve other people, in terms of comparing one's appearance to that of others (10) or asking others for reassurance regarding one's appearance (11). With increasing digitalization, BC nowadays encompasses new types of behaviors such as taking selfies and repeatedly checking one's appearance in them (12), checking one's body parts in photos uploaded to social media [c.f. (13)], participating in social media challenges (e.g., “skinny check”), or comparing one's size, shape, and weight to other digitally posted bodies on social media platforms (14).

While the strategies used to check one's body vary, all have been found to be associated with greater ED pathology (5, 9, 15) such as body dissatisfaction (16), overvaluation of one's weight and shape (17), dietary restriction (18, 19), and negative treatment outcomes (20). Besides its prominence in EDs, studies have also shown that body-related checking is a generally normative behavior (5), which is likewise present in healthy individuals (6) who tend to apply similar BC strategies to those listed above for patients with EDs (7, 21). However, even among healthy females, participants with higher body dissatisfaction reported more BC of disliked body parts compared to participants with a rather positive attitude toward their body (22), suggesting a clinical significance of assessing one's shape, weight, and size through BC also in the non-clinical population.

Although research has demonstrated strong associations of BC with ED symptoms both in patients with EDs and in non-clinical participants, the distinct consequences of BC cannot be clearly derived (23). Generally, BC is classified as a dysfunctional safety behavior (24) and is linked to obsessive-compulsive disorder, in which checking behavior is the most frequently applied compulsion (25), to reduce unease and fear (26, 27). Transferred to body-related checking, BC is postulated to have an anxiolytic function by neutralizing the overconcern with one's shape and weight (9, 28). Adopting this notion, cognitive-behavioral theories of EDs emphasize two different functions of BC: an initial short-term relief from negative emotions (24), followed by a longer-term maintenance of negative emotions and ED pathology (29).

Describing the short-term function of BC, Williamson and colleagues (24) postulated that BC acts in the short term to help undo negative emotions that are triggered by distorted information processing concerning body stimuli (e.g., selective attention on negatively valenced body parts). With regard to the longer-term consequence of BC, Fairburn and colleagues (29) assume that over time, engaging in BC leads to increased arousal, which magnifies the selective attention on assumed bodily imperfections. This selective attention in turn increases the negative affect that the individual was seeking to alleviate in the short term through BC (4), thus reinforcing the patient's belief in the necessity and utility of BC to cope with upcoming negative emotions and to regain a perceived loss of control. Instead, the outcome is that BC itself increases anxiety and strengthens a negative body evaluation over time (24, 29). Through the creation of this vicious cycle, BC is not only activated by ED-specific thought processing (24) but also helps to maintain this way of thinking in the longer term (4).

With respect to the function of BC to reduce negative affect in the short term, only a small number of studies have empirically tested the model assumptions. Kraus and colleagues (30) assessed BC in patients with anorexia nervosa (AN) and bulimia nervosa (BN) in their natural environment by means of ecological momentary assessment. Contrary to the theoretical short-term effect described above, the authors found an increase in negative emotions and a reduction of positive emotions immediately after a BC episode (30). A laboratory study by Shafran et al. (31) in non-clinical women yielded comparable findings: In an experimental induction of BC, women in a “high body checking” condition did not show a reduction in body dissatisfaction, but rather showed increased body dissatisfaction, feelings of fatness, and negative self-critical thinking. However, in an online study by Hartmann and colleagues (32), patients with ED symptoms retrospectively reported a reduction of negative affect from immediately before to after a recalled BC episode. Other retrospective assessments also confirmed the postulated assumptions of reduced negative emotions after BC, with even stronger effects being reported in females with high body dissatisfaction (21, 33).

Hence, studies on the short-term function of BC have yielded inconsistent findings, which may be attributable to different measurement methods between retrospective assessments and assessments before and after actually performed BC. Besides this, the ambivalent results might be due to the different time intervals in which BC was assessed. In most experimental surveys, the emotional state after BC was gathered shortly after the respective BC episode [e.g., (31, 34)]. However, as the time course of BC consequences has not yet been determined, it is unclear whether the study findings describe the short-term relief or whether they already reflect the longer-term maintaining mechanism. For this reason, to the best of our knowledge, no research to date has formed distinct categories regarding the exact amount of time denoted by short-term, longer-term or even long-term effects of BC. Derived from the time course of the research above, we define short-term functions of BC as effects that are directly attributable to one specific BC episode [e.g., (34)], longer-term consequences of BC as effects that are due to multiple, repeated episodes in a defined time frame [e.g., (23)], and long-term consequences of BC as effects that are unlimited in time and occur after habitual performed BC [e.g., (35)].

As outlined above, most previous studies focused on short-term functions of BC. In terms of the longer-term maintenance of negative affect and ED pathology, one study examining longer-term effects (23) and two studies examining long-term effects (35, 36) revealed ambiguous findings. Bailey and Waller (23) examined BC in a 1-day interval in a naturalistic environment: Non-clinical female participants measured their wrist size every 15 min for 8 h on 1 day, and did not check the following day, in a randomized order. Contrary to the model assumption, the authors found no difference between the two conditions in terms of body dissatisfaction or general eating attitudes, with the exception of an increase in the fear of uncontrollable weight gain in the BC condition. Likewise, regarding long-term effects, using ecological momentary assessment, Sala et al. (36) found that BC did not predict any ED pathology, such as body dissatisfaction or drive for thinness, in a sample diagnosed with EDs. By contrast, the other study focusing on long-term consequences, conducted by Zaitshoff et al. (35), found that typical engagement in BC predicted ED pathology 3 to 4 months later in a sample of college students, which is consistent with the proposed pathology-maintaining effect of BC. However, in contrast to the study by Sala et al. (36), the latter study relied on self-reported questionnaire data, and participants were not actually asked to check their body.

As evident from the reported literature, findings regarding short-term, longer-term, and long-term consequences of BC are inconsistent. However, the largest research gap can be found in the area of longer-term consequences. In the aforementioned study by Bailey and Waller (23), the engagement in BC was experimentally restricted as the authors only focused on one fixed BC behavior (i.e., checking wrist size), thus neglecting naturally performed BC behaviors. Furthermore, as previous studies have focused on ED symptoms, the proposed longer-term maintaining consequence of increased negative affect (24) has not yet been investigated.

Generally speaking, besides the effect of BC on ED symptoms and negative affect, subsidiary functions of BC have mainly been disregarded in previous research. However, BC is not the only strategy that presumably helps persons with ED to “undo” distress or that influences ED symptoms (9, 10). Patients with EDs show other compensatory strategies to cope with the fear of weight gain, such as purging in BN and restrictive eating in AN (4, 24). Moreover, some patients show the counterpart of body checking by completely avoiding confrontation with their shape or weight, namely *body avoidance* (5). Therefore, certain functions must be inherent to BC such that the behavior is maintained and is not replaced by other strategies that also aim to reduce negative affect. In a first analysis of individuals with a self-reported diagnosis of one of the mental disorders in which BC is usually performed (see above), participants rated their negative affect and their endorsement of functions of BC (32). Attainment of certainty was found to be the most relevant function. In particular, in those participants with a self-reported body image disorder (i.e., AN, BN, BDD), a characteristic function of BC was self-motivation (32). So far, data only exist on symptomatic women, even though non-clinical individuals also tend to perform BC on a daily basis (5). In healthy individuals, BC might serve different functions, as it assumed that in contrast to patients with EDs, they do not experience an attentional bias toward disliked body parts (37). Extensive functions of BC have not yet been examined in non-clinical participants, especially using longer-term assessments.

Based on the inconsistent findings on the functions and consequences of BC, the present online study empirically examined the postulated longer-term negative consequences of BC. Hence, we compared the impact of an experimentally increased frequency of BC (experimental condition) with the idiosyncratic typical amount of BC (control condition) within the daily life of non-clinical women. Participants took part in both conditions using a crossover design, with each condition running for 3 days. In order to identify the tipping point between the theoretically assumed functional short-term and dysfunctional longer-term BC (29), the first objective of the study was to examine the proposed longer-term consequences. We therefore hypothesized that in both conditions, BC episodes would lead to a longer-term increase in a trait-like battery of negative affect, in ED symptoms, and in general pathology (I).

The second objective was to identify distinct features of the (dys)functionality of BC in terms of the quantitative amount of BC. Based on the aforementioned findings by Bailey and Waller (23), we expected to find a stronger deterioration in the trait-like battery (see above) in the experimental condition than in the control condition (II).

The third and fourth objectives were to foster the understanding of the self-perceived functions of typical BC in non-clinical individuals, as previous studies only examined the different functions of BC in self-diagnosed patients in the short term. In line with Hartmann and colleagues (32), we also expected attainment of certainty and motivation to be the most relevant longer-term consequence of BC in non-clinical individuals (III). In addition, Wilhelm and colleagues (21) found that participants who endorsed a higher number of BC strategies generally showed greater body dissatisfaction. In the present study, we tested whether these findings can be transferred to the different BC functions. Thus, we assumed that a higher number of endorsed functions of BC would result in a higher likelihood of engaging in BC. Specifically, we hypothesized that a higher number of BC functions endorsed in preliminary questionnaires would predict increased body dissatisfaction, negative affect, and the amount of typical BC in the longer term, that is after an episode of typical BC (IV).

## Materials and Methods

### Participants

**Figure 1:**
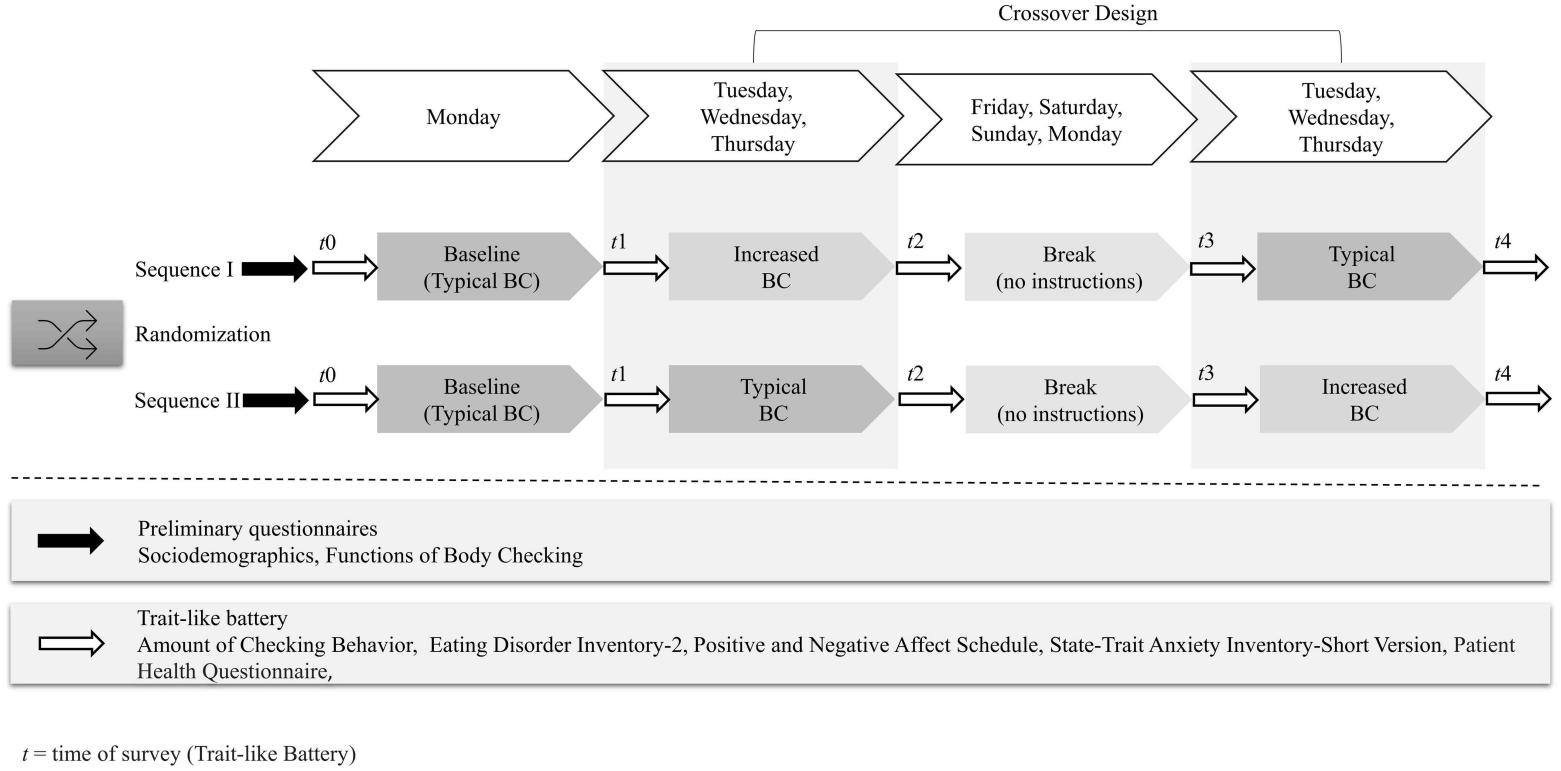
Procedure of the study.

As inclusion criteria, participants needed to identify as female, be aged between 18 and 65 years, and report no current or past mental disorders. Exclusion criteria were suicidal or self-harm behavior, acute intoxication by psychotropic substances or the use of psychiatric drugs. A total of *N* = 427 college students clicked on the link provided through the University mailing list, of whom *n* = 324 began the survey (75.8 %). Over the five time points of the survey (see Figure 1), a total of 138 participants dropped out. Of these, *n* = 60 dropped out at the first survey time point, *n* = 20 at the second, *n* = 18 at the third, *n* = 20 at the fourth, and *n* = 20 at the fifth time point. As the highest dropout rates occurred at the landing page for each time point (*t*0–*t*4), no systematic effects for dropout are assumed. Prior to the analysis, *n* = 16 data sets were excluded due to double participation. After completing the preliminary questionnaires, three participants were excluded as they did not meet the study criteria (*n* = 1 due to male sex; *n* = 2 due to the use of antidepressants). Hence, the final sample comprised *n* = 167 (39.1 %) females. Participants received course credit as reimbursement. The study was approved by the ethics committee of Osnabrueck University.

### Measures

#### Assessment of Body Checking Behavior

On each separate day of the two 3-day conditions, participants self-reported the amount of performed BC by using a multicounter application on their smartphone. Participants were instructed to increase the counter by plus one (+1) each time they had performed BC in the time from 8:00 am until 8:00 pm and to start a new counter for each of the 7 days of assessment. After each 3-day episode, they received an E-mail invitation to enter the number of their daily BC on an online survey platform.

#### Functions of Checking Questionnaire

To assess the purpose of typical BC in non-clinical individuals, in a preliminary questionnaire prior to the experiment, the participants rated their endorsement of five specific functions of BC in the Functions of Checking Questionnaire (32) on a 5-point Likert scale from 1 = *not at all* to 5 = *very much*. The items comprise the following functions of BC: *Avoidance of Negative Consequences, Further Motivation, Estimating the Possibility of Concerns, Attainment of Certainty, Achievement of Control*. Following Hartmann et al. (32), the analyses were run on a single-item level. Therefore, no internal consistency score is provided. Moreover, due to the novelty of this questionnaire, we further cannot provide test-retest reliabilities from other studies.

#### Eating Disorder Inventory-2

The Eating Disorder Inventory-2 [EDI-2 (38, 39)] is a self-report questionnaire assessing ED pathology. In the present study, the following two subscales were used: The *Body Dissatisfaction* subscale assesses the degree of (dis)satisfaction with one's shape, abdomen, hips, buttocks, and thighs using nine items. The *Drive for Thinness* subscale assesses the desire to lose body fat and the preoccupation with one's own body. Items from both subscales are rated on a 6-point Likert scale ranging from 1 = *never* to 6 = *always*, with higher scores indicating greater symptom severity. In the present study, participants were asked to refer to the past 3 days (instead of the usual 28 days) in their responses, in order to cover potential changes in subjective body dissatisfaction due to the respective BC episode. In previous studies in healthy females, both subscales have shown excellent to nearly acceptable internal consistencies [*Body Dissatisfaction* α = 0.90; (40); *Drive for Thinness* α > 0.58; (41)]. In the present study, Cronbach's α can be rated as excellent for *Body Dissatisfaction* (α = 0.99) and for *Drive for Thinness* Scale (α = 0.98) at all time points. The test-retest reliability of the EDI-2 over a 7-day interval ranges from *r*_tt_ = 0.86 until *r*_tt_ = 0.89 (39).

#### Positive and Negative Affect Schedule

To evaluate the theoretically proposed longer-term consequences of BC on affect due to BC, we applied the self-report Positive and Negative Affect Schedule [PANAS (42, 43)]. As the instructions can vary according to study purposes (43), we asked the participants to consider the last 3 days when completing the PANAS, which corresponds to one BC episode. The PANAS encompasses 20 items as single adjectives that describe different perceptions and feelings, with 10 items corresponding to the broad dimension of *Negative Affect* and 10 items corresponding to *Positive Affect*. We utilized both dimensions, as previous studies on BC assumed that negative affect does not necessarily equate to a lack of positive affect [e.g., (30)]. The items are rated on a 5-point Likert scale ranging from 1 = *not at all* to 5 = *extremely*. The PANAS has proven to be highly internal consistent for both subscales [*Positive Affect*: α = 0.88; *Negative Affect*: α = 0.85; (43)]. In the present study, internal consistency was likewise good (*Positive Affect*: α = 0.88; *Negative Affect*: α = 0.91). The test-retest reliability of the PANAS over an interval of 8 weeks was *r*_tt_ ≤ 0.66 (42).

#### State-Trait Anxiety Inventory Short Version

Research has shown that a higher degree of BC is positively associated with anxiety in ED patients and non-clinical individuals (44). To retest this finding in the case of experimentally increased BC and typical BC, we assessed emotional anxiety in more detail using the one-dimensional State-Trait Anxiety Inventory Short Version [STAI-SKD (45)]. To cover anxiety within each BC episode, we instructed participants to reflect upon the last 3 days. The instrument comprises 5 items that are rated on a 4-point Likert scale ranging from 1 = *not at all* to 4 = *very much*. As in other research reporting good internal consistency [α = 0.81 (32)], internal consistency was good in our sample (α = 0.89 for all time points). Furthermore, no test-retest reliability is provided for the short version of the STAI. However, for the full version of the STAI, *r*_tt_ = 0.97 was reported for trait anxiety and *r*_tt_ = 0.45 for state anxiety in a student sample over an interval of 21 days (46).

#### Patient Health Questionnaire

The Patient Health Questionnaire [PHQ-9 (47, 48)] is a one-dimensional self-report screening instrument for major depressive disorder, which served as a measure of general pathology in the present study. In order to examine general pathology during BC episodes, we asked participants to respond to the 9 items referring to the last 3 days (instead of the usual 2 weeks). Items are rated on a 4-point Likert scale ranging from 0 = *not at all* to 3 = *nearly every day*. Internal consistencies in a non-clinical student sample were good [α = 0.85 (49)]. In the present sample, internal consistencies can be rated as excellent (α = 0.96 for all time points). A good test-retest reliability (*r*_tt_ = 0.89) was shown in the same non-clinical student sample reported above over an interval of 1 month (49).

### Experimental Conditions

In a cross-over design (see Figure 1), participants completed two conditions (experimental and control condition), each lasting for 3 successive days. To avoid order bias, the order of each sequence (experimental or control condition first) was randomized. In the experimental condition, participants were asked to check their body at a 3-fold increased frequency, using their typical amount of baseline BC as a reference. In the control condition, participants were asked to stick to their typical BC. The first condition was followed by 4 days without guidance to avoid carry-over effects. Subsequently, participants underwent the task on the same days of the week as the previous condition. At the beginning of each condition, participants received a similar definition of BC, explaining that it entails the repeated inspection of one's body (1) in order to obtain information about size, weight and shape (4). Furthermore, with each condition, participants were asked to reflect upon and then perform their own natural, typical BC behavior, meaning that they were not given specific instructions on what to check on their body. For the purpose of inspiration only, they were given the following examples of BC: “weighing, mirror gazing, pinching body parts (9), comparing one's shape to others (10) or to posts including bodies on social media platforms (14).” After providing the definition and describing strategies, in the experimental condition, the instructions were phrased as follows: “We would like you to triple your BC for the next days (Tuesday to Thursday) and to count it separately for each day.” In the control condition, we changed the instructions with regard to the amount of BC: “We would like you to stick to your typical BC behavior on a regular basis […]”.

### Procedure

The data collection period of the online experiment covered 7 days, with an additional break of 4 days (“washing out”) between the respective conditions. Once participants provided informed consent to the study goals and were informed of the voluntary nature of participation and the anonymized data storage, they were randomly assigned to one of the two sequence orders (see study procedure in Figure 1). To capture BC in daily life, the study began on a Monday for each participant. First, in preliminary questionnaires, the participants learned the definition and examples of BC (see above), provided demographic (age, gender, nationality), body-related (weight, height), and psychological data (use of drugs, physical illness, current or past therapeutic treatment), and indicated their endorsement of different functions of BC with the Functions of Checking Questionnaire (32). Following this, they completed the trait-like battery for the first time (*t*0), measuring ED symptoms with the EDI-2 (38, 39), affect with the PANAS (42, 43) and STAI-SKD (45), and general pathology with the PHQ (47, 48). Participants were then asked to complete a 1-day baseline from Monday until Tuesday by recording their usual daily amount of BC in a smartphone-based counting app. Next, on Tuesday, they were asked about the amount of their baseline BC from the day before, the trait-like battery was again administered (*t*1), and the participants started with one of the two BC conditions of typical BC or increased BC that lasted for 3 days each from Tuesday until Thursday. Additionally, they received an E-mail with a reminder to stick to their individual BC rate for the respective condition (baseline amount for typical BC and tripled baseline amount for increased BC). Before and after each 3-day episode of BC, subjects received an E-mail with a link to a survey in which they were asked to enter their daily recorded amount of BC for each of the 3 days separately and to again complete the trait-like battery (*t*2–*t*4).

### Data Analysis

The statistical analyses were run using the software IBM Statistical Package for the Social Sciences [version 26; IBM, (50)], with the exception of the manipulation check of equivalence between the baseline and the typical BC condition, which was performed using jamovi [version 1.6 (51)]. In a first step, sociodemographic and health-related data were merged to *M* (*SD)* and *Range*. To ensure a successful manipulation, we conducted 4 manipulation checks: First, the 2 sequences of typical and increased BC were tested for selection bias using analyses of variance (ANOVAs) with Bonferroni correction, with the within-subjects factor Condition (i.e., experimental, control) and the between-subjects factor Sequence (i.e., first typical BC or first increased BC). Second, *t*-tests for dependent samples were conducted to ensure the successful manipulation that participants actually increased their BC in the respective experimental condition. Third, to check for sequence effects, we conducted an ANOVA with the independent variable Sequence and the dependent variable Amount of BC. Fourth, to test for equivalence between the amount of BC at baseline and the amount of BC in the typical BC condition, two one-sided tests (i.e., TOST procedure) were used following Lakens et al. (52). The TOST procedure was calculated against the equivalence bounds stemming from the smallest effect size of interest of Cohen's *d* = 0.5, which is proposed by Norman et al. (53) for health-related outcomes. Equivalence between baseline BC and typical BC can be assumed if both of the one-sided *t-*tests for dependent samples are considered statistically significant (52).

To test the influence of BC on the trait-like battery as proposed in hypotheses I and II, we used repeated measures (multivariate) analyses of variance (rm-[M]ANOVAs) in a 2 × 2 design with the within-subjects factors Time (i.e., pre, post each checking episode) and Condition. To test for mean differences between typical BC and increased BC and for mean differences between pre and post 3-day BC episodes, we used single rm-MANOVAs for multifactorial measures, that is the dependent variables of ED pathology (EDI-2), and rm-ANOVAS for one-dimensional measures as the dependent variables of emotion (STAI-SKD, PANAS) and general pathology (PHQ-9). For the rm-MANOVAs, any significant effects were broken down into single rm-ANOVAs followed by pairwise comparisons. If the assumption of sphericity was not met for the dependent factors (*p* < 0.05 for Mauchly's test of sphericity), Greenhouse-Geisser-corrected degrees of freedom were applied. Effect sizes for the factor differences and interactions were reported using partial eta-squared ($\eta_{\text{p}}^{2}$). In accordance with Cohen (54), values are classified as small (0.01), moderate (0.06), and large (0.14). Regarding the functions of BC in hypothesis III, we descriptively examined *M* (*SD*) of single functions. To test the influence of the number of endorsed functions of BC in hypothesis IV, we used linear regression analyses with the number of functions assessed in preliminary questionnaires as the dependent variable and body dissatisfaction, negative affect, and the amount of BC as independent variables assessed after the typical BC episode.

## Results

### Participant Characteristics

Participant characteristics are provided in Table 1, which reveals a mean BMI in the normal weight range (*Range* 18–34) and a mean age in the early twenties (*Range* 18–53). None of the participants exceeded the cut-off scores of the trait-like battery, which would have indicated a clinically severe level of symptoms, reflecting a level of body dissatisfaction in the sample which lies within the usual range for non-clinical subjects (55). Overall, the low prevalence of clinical symptoms indicates a healthy sample without eating disturbances.

**Table 1:** Means and standard deviations of demographic characteristics and trait-like measures.

**Variable**	**Total sample**		**Sequence 1** ^a^		**Sequence 2** ^b^		**Statistics**
	**(*N* = 167)**		**(*n* = 83)**		**(*n* = 78)**		
	***M***	***SD***	***M***	***SD***	***M***	***SD***	
Age	23.8	6.5	23.3	5.7	23.9	6.6	*F*_(1, 159)_ = 0.37, *p* = 0.542
BMI^c^	23.3	10.7	23.9	14.2	22.7	5.4	*F*_(1, 159)_ = 0.52, *p* = 0.472
EDI-2 DT^d^	2.8	1.5	3.0	0.9	2.9	0.9	*F*_(1, 159)_ = 0.09, *p* = 0.766
EDI-2 BD^e^	2.9	1.3	3.1	0.4	3.0	0.4	*F*_(1, 159)_ = 0.79, *p* = 0.375
STAI-SKD^f^	1.5	1.3	1.6	0.6	1.7	0.6	*F*_(1, 159)_ = 1.33, *p* = 0.251
PANAS pos^g^	2.4	1.8	2.7	0.7	2.8	0.6	*F*_(1, 159)_ = 0.93, *p* = 0.335
PANAS neg^h^	1.2	1.8	1.4	0.4	1.4	0.6	*F*_(1, 159)_ = 1.13, *p* = 0.289
PHQ-9^i^	0.7	0.4	0.7	0.5	0.8	0.5	*F*_(1, 159)_ = 0.52, *p* = 0.472

*To control for order effects, Statistics show results of the ANOVA between the two Sequences*.

a *First typical, then increased body checking*.

b *First increased, then typical body checking*.

c *Body Mass Index*.

d *Eating Disorder Inventory-2, subscale Drive for Thinness*.

e *Eating Disorder Inventory-2, subscale Body Dissatisfaction*.

f *State-Trait Anxiety Inventory Short Version*.

g *Positive and Negative Affect Schedule, subscale Positive Affect*.

h *Positive and Negative Affect Schedule, subscale Negative Affect*.

i *Patient Health Questionnaire-9*.

### Manipulation Checks

The manipulation was considered as successful. First, to rule out selection bias, regarding the equal distribution of participants across the two sequences (typical BC first or increased BC first), the ANOVAs showed no differences in the amount of typical BC at baseline [*F*_(1, 158)_ = 0.734, *p* = 0.393], in participants' sociodemographic characteristics, or on the trait-like battery (see Table 1). Second, regarding the amount of BC averaged over 3 days, the *t*-test for dependent samples indicated that participants performed BC significantly more often during the experimental condition than during the control condition [*t*_(144)_ = 9.95, *p* < 0.001]. However, while participants were asked to triple their BC in the experimental condition, mean comparisons indicated that they only doubled their amount of BC (typical BC: *M* = 9.0, *SD* = 10.1; increased BC: *M* = 17.0, *SD* = 15.3). Table 2 shows the daily amount of BC in the typical BC and the increased BC condition. Third, there were no differences between the two conditions as a function of sequence for the typical BC [*F*_(1, 150)_ = 0.893, *p* = 0.346] or increased BC condition [*F*_(1, 148)_ = 0.632, *p* = 0.428]. Fourth, with respect to the test for equivalence between baseline and typical BC, the two one-sided *t-*tests were significant for the upper bound [Δ_*U*_: *t* = −7.09 (149), *p* < 0.001] and for the lower bound [Δ_*L*_: *t* = 5.16 (149), *p* < 0.001], depicting a 90 % CI [−1.19, 0.31], which lay between the bounds of −2.77 and 2.77 on a raw scale (*d* = −0.5–0.5). Therefore, baseline BC and typical BC can be considered as statistically equivalent. Exact BC rates of the experimental conditions are given in Table 2, and the checking rate for all participants from the 1-day baseline condition prior to the experimental manipulation lay at *M* = 8.9 times (*SD* = 12.5).

**Table 2:** Amount of checking in the typical checking and increased body checking condition.

**Condition**	**Total sample**				**Sequence 1^a^**				**Sequence 2^b^**			
	**Typical^d^**		**Increased^e^**		**Typical^d^**		**Increased^e^**		**Increased^e^**		**Typical^d^**	
	***M***	**SD**	***M***	***SD***	***M***	***SD***	***M***	***SD***	***M***	***SD***	***M***	***SD***
**Day**												
1	8.8	7.3	19.0	23.4	9.1	8.1	19.1	23.9	16.6	14.1	10.1	13.4
2	8.9	10.6	17.9	18.1	8.4	7.0	19.7	23.7	16.9	14.2	9.3	12.0
3	9.5	12.1	16.8	14.8	7.6	6.8	19.3	21.3	16.8	16.1	9.7	12.5
Day 1–3	9.0	10.0	18.0	19.0	8.4	6.8	19.4	22.6	16.8	14.4	9.7	12.4

a *First typical, then increased body checking*.

b *First increased, then typical body checking. ^c^Body Checking Condition*.

d *Typical Body Checking Condition*.

e *Increased Body Checking Condition*.

### Hypothesis I and II: Effects Before and After Checking Episodes in Typical vs. Increased Body Checking

For the following results of the trait-like battery on ED symptoms, affect, and general pathology, *M (SD)* for the two conditions of typical BC and increased BC are presented in Table 3. All found main effects were of small effect size (54).

**Table 3:** Means and standard deviations for the respective conditions.

**Condition**	**Time**		**EDI-2^a^**		**STAI-SKD^d^**	**PANAS^e^**		**PHQ-9^f^**
			**BD^b^**	**DT^c^**		**Positive affect**	**Negative affect**	
Preliminary measures		*M*	3.10	2.90	1.68	2.71	1.39	0.76
		*SD*	0.46	1.10	0.57	0.68	0.48	0.48
Typical checking	Pre BC^g^	*M*	3.16	2.62	1.67	2.54	1.44	0.74
		*SD*	0.96	0.98	0.64	0.84	0.62	0.45
	Post BC^g^	*M*	3.18	2.61	1.54	2.56	1.54	0.70
		*SD*	1.00	0.96	0.59	0.81	0.43	0.47
Increased checking	Pre BC^g^	*M*	3.16	2.66	1.69	2.67	1.39	0.66
		*SD*	0.99	0.94	0.57	0.88	0.54	0.43
	Post BC^g^	*M*	3.19	2.63	1.58	2.60	1.53	0.69
		*SD*	1.02	0.82	0.58	0.87	0.44	0.42

a *Eating Disorder Inventory-2,*

b *Subscale Drive for Thinness,*

c *Subscale Body Dissatisfaction*.

d *State-Trait Anxiety Inventory Short Version*.

e *Positive and Negative Affect Schedule, subscale Positive Affect and subscale Negative Affect*.

f *Patient Health Questionnaire-9*.

g *Body Checking*.

#### Eating Disorder Symptoms

No effects of BC on ED symptoms emerged in the present sample. When examining the EDI-2 subscales, the two-way interaction Time × Condition was not significant [Λ = 0.99, *F*_(2, 135)_ = 0.78, *p* = 0.461, $\eta_{\text{p}}^{2}$ = 0.01], nor were the main effects of Time [Λ = 0.97, *F*_(2, 135)_ = 1.73, *p* = 0.181, $\eta_{\text{p}}^{2}$ = 0.03] and Condition [Λ = 0.97, *F*_(2, 135)_ = 1.96, *p* = 0.144, $\eta_{\text{p}}^{2}$ = 0.03].

#### Affect

Regarding the emotional states of *Positive Affect* and *Negative Affect* within the PANAS, the two-way interaction Time × Condition [Λ = 0.97, *F*_(2, 134)_ = 2.08, *p* = 0.129, $\eta_{\text{p}}^{2}$ = 0.03] did not reach significance. However, the results revealed a significant main effect of Time [Λ = 0.95, *F*_(2, 134)_ = 1.85, *p* = 0.043, $\eta_{\text{p}}^{2}$ = 0.05], but no significant main effect of Condition [Λ = 0.99, *F*_(2, 134)_ = 0.57, *p* = 0.570, $\eta_{\text{p}}^{2}$ = 0.01]. With regard to the factor Time, separate univariate *post-hoc* ANOVAs revealed significant main effects only for the subscale *Negative Affect* [*F*_(1, 135)_ = 5.45, *p* = 0.021, $\eta_{\text{p}}^{2}$ = 0.04] but not for the subscale *Positive Affect* [*F*_(1, 135)_ = 1.49, *p* = 0.224, $\eta_{\text{p}}^{2}$ = 0.01]. The mean values for *Negative Affect* were higher after each condition than before each condition, indicating an increase in longer-term negative affect after each 3-day BC episode. Regarding anxiety in the STAI-SKD, the interaction of Time × Condition was not statistically significant [*F*_(1, 136)_ = 1.21, *p* = 0.273, $\eta_{\text{p}}^{2}$ = 0.01]. However, the 2 × 2 rm-ANOVA yielded significant main effects of Time [*F*_(1, 136)_ = 4.45, *p* = 0.037, $\eta_{\text{p}}^{2}$ = 0.03] and Condition [*F*_(1, 136)_ = 5.39, *p* = 0.022, $\eta_{\text{p}}^{2}$ = 0.04]. *Post hoc* analyses revealed that the mean values for Time were lower after each BC episode than before each episode, suggesting a decrease in anxiety after each 3-day episode of BC. Moreover, a main effect of Condition emerged, insofar as participants in the increased BC condition generally showed less longer-term anxiety. Taken together, after a 3-day episode of BC, *Negative Affect* increased and anxiety decreased.

#### General Pathology

For changes in general pathology due to BC, the 2 × 2 rm-ANOVA for the PHQ-9 showed a significant interaction effect of Time × Condition [*F*_(1, 136)_ = 0.97, *p* = 0.031, $\eta_{\text{p}}^{2}$ = 0.03], revealing the pattern of an ordinal interaction: While participants in the increased BC condition showed increased general pathology, those in the typical checking condition showed fewer of these symptoms. Nevertheless, we found no main effects of Time [*F*_(1, 136)_ = 0.20, *p* = 0.658, $\eta_{\text{p}}^{2}$ = 0.00] or Condition [*F*_(1, 136)_ = 0.98, *p* = 0.113, $\eta_{\text{p}}^{2}$ = 0.02] in terms of general pathology.

### Hypothesis III and IV: Functions of Checking

Regarding the endorsement of different functions of typical BC assessed in preliminary questionnaires, participants most often endorsed the function of attainment of certainty, followed by motivation, and the attainment of control. Exact means regarding the frequency of endorsement of all given BC functions are presented in Table 4. Regarding the number of endorsed functions of BC as a predictor of longer-term negative affect assessed after the 3-day typical BC episode, the multiple correlation of *R* = 0.25 for the amount of typical BC and body dissatisfaction was found to be statistically significant [*F*_(1, 165)_ = 10.88, *p* < 0.001]. However, endorsement of a higher number of different BC functions did not lead to higher body dissatisfaction on the EDI-2 subscale (ß = 1.62, *p* = 0.661), but did significantly predict higher negative affect on the PANAS (ß = 0.249, *p* < 0.001) and a greater amount of typical BC (ß = 0.261, *p* < 0.001).

**Table 4:** Means (*M*) and standard (*SD*) Deviations of Functions of Body Checking.

**Function of checking item**	**Frequency *(N* = 167*)***	***SD***
Avoidance of negative consequences	2.1	0.1
Further motivation	2.4	1.0
Estimating the possibility of concerns	2.1	0.9
Attainment of certainty	2.5	1.0
Achievement of control	2.3	1.0

## Discussion

While many studies have investigated the short-term function of BC, that is to reduce negative affect, the proposed longer-term consequences of BC, to maintain negative affect and ED pathology (24, 29), have rarely been examined. Therefore, to confirm the longer-term maintaining consequence of BC, that is to increase negative emotions and eating-related cognitions, the present study investigated the course of affect, ED symptoms, and general pathology in non-clinical women over two 3-day BC episodes of unmanipulated typical BC and experimentally induced increased BC. Additionally, we were interested in further self-perceived functions of typical BC and in the extent to which the endorsement of a higher number of these functions predicted higher longer-term negative affect.

First, we hypothesized that in the longer term, engagement in BC would lead to an increase in negative affect, ED symptoms, and general pathology irrespective of the condition (i.e., typical BC or increased BC). As predicted, the performance of BC resulted in increased negative affect at the end of each 3-day BC episode compared to baseline, suggesting that BC behavior generally produces aversive emotions in the longer term. This is in accordance with theoretical assumptions of ED models [cf. (24)], which state that although engagement in BC relieves negative affect in the short term, it contributes to the maintenance of negative affect in the longer term. Contrary to expectation, anxiety, as a facet of negative affect, was lower in the longer term after an episode of BC compared with before the episode. This is in contrast to theoretical assumptions of a short-term decrease and a longer-term increase in negative emotions, which generally include anxiety. This discrepancy might be attributable to potentially different consequences in terms of anxiety between non-clinical individuals and patients with EDs. Studies in patients with EDs have shown that by engaging in BC, clinical samples become sensitized to the fear of uncontrollable weight gain (1, 56), likely because BC increases selective attention (57) toward disliked body parts (58). In turn, participants in the present non-clinical sample may have become habituated, rather than sensitized, to potential fear that is evoked by BC, as they do not experience self-devaluing thoughts (59, 60) and selective attention (37, 60) and show a rather balanced viewing pattern during BC (5, 61, 62). Thus, regarding the association between BC and affect overall, the present results suggest that although BC reduces anxiety for non-clinical subjects over time, the uncomfortable process of BC leads to a generally negative affect. Despite the ambiguity of our findings regarding anxiety, the longer-term increase in overall negative affect does indicate the predictive pathological value of BC, as proposed in etiological models [e.g., (29)].

Moreover, also contrary to expectation, we found no differences in ED symptoms between the beginning and end of a BC episode. Thus, participants' performance of BC did not lead to a longer-term change in concerns about size, weight, and shape. As such, our findings are not in line with theoretical assumptions that BC contributes to ED symptoms in the longer term (29). Likewise, the present results do not correspond to other non-clinical research on short-term functions of BC, which reported a strong positive association between BC and eating pathology regarding ED symptoms (6), feelings of fatness (31), or body dissatisfaction (63, 64). Unlike the aforementioned research on short-term functions of BC that suggest an influence of BC on ED pathology, the one other study that examined longer-term consequences, by Bailey and Waller (23), also found no changes in weight and shape concerns in non-clinical women as a consequence of BC. Longer-term consequences of BC on the maintenance of ED pathology have been theoretically proposed (24, 29) but are yet to be empirically confirmed. Similarly, with regard to long-term consequences of BC, in an ecological momentary assessment study, Sala et al. (36) did not find that BC predicted ED pathology such as future bulimic symptoms, drive for thinness, or body dissatisfaction in a sample diagnosed with an ED. This suggests that the absence of effects on ED pathology that is shown in the longer term also continues in the long term.

While an integration of our longer-term findings into research that deviates from theory raises the question of consistency, a potential pattern emerges when examining the time ranges of measurements: In studies that reported short-term associations between BC and eating pathology, the changes were short-lived (18) and subsided after 10 (64), 15 (21), or 30 min (31). In the longer term and the long term, in studies in which participants actually checked their body in a naturalistic setting, no associations between BC and eating pathology were found 1 day (23), 3 days (i.e., our study), or 1 month (36) after BC episodes. Potentially, the influence of BC on ED symptoms therefore subsides in the longer term. Nonetheless, although no effects on ED symptoms emerged, the present results did reveal that BC increased negative affect. Therefore, the impact on eating pathology might be more time-sensitive than the impact on affect [cf. (65)]. The discrepancy between our findings and ED theory highlights the need for further research on the longer-term consequences of BC in order to clarify the impact of BC on ED symptoms.

Our second hypothesis examined the impact of frequency on BC. Specifically, we expected negative affect, ED symptoms, and general pathology to be higher in the experimental condition of increased BC than in the control condition of typical BC. While there were no differences between conditions regarding negative affect or ED symptoms, anxiety was even found to be lower in the increased BC than in the typical BC condition. This lends further credibility to the results from hypothesis I, indicating that BC has an anxiolytic function in non-clinical subjects in the longer term. Moreover, BC impacted general pathology insofar as the experimental condition was associated with greater general pathology. This corresponds to theoretical models on EDs which assume that a higher frequency of BC leads to a higher likelihood of ED maintenance (4). Our findings on the negative consequences of increased BC are complemented by previous research reported that a higher amount of BC negatively influences general mental health and quality of life (66) as well as symptoms of ED (5, 18). Further highlighting the pathological impact of a higher frequency of BC, questionnaire-based (7, 67) and retrospective (32) studies found that BC frequency was lower in non-clinical individuals than in patients with EDs, especially BN (7). Combined with the negative impact on increased BC found in our non-clinical sample, this leads to the assumption that the amount of BC can be taken as a broad indicator of the pathological significance of BC.

Besides the negative consequences of increased checking, we found an unexpected decrease in general pathology in the control condition, indicating that our non-clinical participants seem to have benefited from typical BC. This finding is in contrast to theoretical assumptions defining BC as a dysfunctional behavior that, regardless of frequency, contributes to the maintenance of EDs [(68), p. 104]. However, the positive impact of typical BC on general pathology found in the present study may stand to reason when considering BC as dimensional and when referring to the second safety behavior, namely body avoidance (28). Often, avoidance and checking are regarded as two opposite poles of safety behavior (5, 18) that may fluctuate and alternate (4) and that are therefore both theoretically regarded as disorder-maintaining (5). On the one hand, the mechanism of avoiding looking at certain body parts is seen as preventing confrontation with reality (27). On the other hand, the mechanism of increased checking is considered to magnify perceived imperfections (4). Consequently, along with increased BC, body avoidance is likewise regarded as dysfunctional. In view of the harmful nature of both high body avoidance and high BC, the present results on the potential helpfulness of typical BC suggest a “just right” amount, indicating that a healthy amount of typical BC might be a protective factor in the longer run.

It is therefore possible that our non-clinical participants naturally tended to check only up to their healthy limit, but not beyond this, which might explain why they only doubled their checking in the increased BC condition even though they were tasked to triple it. If replicated, this “just right” amount of BC may thus be a distinguishing factor between non-clinical and clinical individuals, according to which patients with EDs may have lost their sense of healthy typical BC. The assumed existence of a non-harmful amount of BC might further explain why non-clinical individuals also frequently scrutinize their bodies without developing clinical pathology (6, 9). Still, the assumed inverted U-shaped association of BC is not compatible with ED models and needs to be replicated and further examined by including clinical samples.

As expected in our third hypothesis, the attainment of certainty and motivation were the functions of highest relevance for our non-clinical population. This is in line with findings in patients with ED symptoms reported by Hartmann et al. (32). In the latter study, the authors reasoned that the importance of attainment of certainty was explained by the fact that patients with EDs often experience an intolerance of uncertainty. The concept of intolerance of uncertainty is related to checking (69) and indicates that a situation (e.g., not knowing how one looks in a mirror) is difficult to endure (70, 71), leading to reassurance behavior such as BC. However, intolerance of uncertainty likewise exists in healthy individuals (72–74). While our non-clinical women also seemed to engage in BC in order to attain certainty, intolerance of uncertainty might again be the leading motivator for the craving for certainty in this sample. Given that both patients with ED and non-clinical women seem to strive to attain certainty, this function might be less of a distinguishing factor between the two groups. Instead, irrespective of population, intolerance of uncertainty might act as a negative reinforcer of BC, as is assumed by the theoretical models with respect to negative affect (29).

Likewise, in line with findings from patients with ED symptoms (32), to further motivate oneself was a second important reason for engagement in BC in our sample. However, motivation might be construed differently in non-clinical individuals and patients with EDs. In the clinical population, patients often pursue ambitious objectives regarding shape and weight (75), meaning that they might need BC to further motivate themselves to hold on to rather unintuitive compensatory strategies (e.g., restrictive eating). In our non-clinical subjects, by contrast, the motivation to check one's body might not originate from a dysfunctional pattern. In the case of a less pronounced drive for thinness (76, 77) and a lower attentional bias toward unattractive body parts (78), BC might motivate healthy individuals to keep on liking their body or to adopt a more balanced lifestyle through realistic feedback rather than further weight loss. Again, regarding the function of motivation from a non-pathological perspective, BC could be rather a protective factor for non-clinical women in the longer term.

In line with our fourth hypothesis, the endorsement of a greater number of BC functions predicted negative affect and the amount of typical BC, indicating that the number of functions of BC impacted mood and the typical rate of BC. This result might be interpreted in light of the findings of Wilhelm and colleagues (21), who reported that when participants endorsed a higher number of BC strategies, they tended to show greater body dissatisfaction. While our goal was to transfer the results found for BC strategies to BC functions, the present results support the suggestion that not only does an increased number of BC strategies lead to body dissatisfaction (21), but that an increased number of functions fulfilled by BC may also lead to negative affect. Yet, according to the present results from hypotheses III and IV, our non-clinical participants did not endorse different types of functions of BC than patients with EDs [cf. (32)]. However, the present results revealed that a higher number of endorsed functions of BC led to greater negative affect. It might be suggested that the reasons for and causes of BC may be less relevant than the amount of BC, which fosters the maintenance of ED pathology.

The present study was the first to examine longer-term consequences of BC in non-clinical females in a natural environment. Nevertheless, some limitations should be mentioned when interpreting the results. First, we did not monitor the participants' compliance with the BC instructions. As our BC conditions were implemented in participants' daily life and relied on self-disclosure, we were unable to verify whether BC was actually performed and if so, whether participants recorded it every time. Moreover, even if participants did perform BC as instructed, we still cannot rule out that they partially avoided checking their least-liked body parts and only concentrated on the liked ones. Furthermore, there is a risk of recall bias, as participants only rated their emotions every 3 days, after the interventions. However, this possibility may have been decreased by the real-time app-based tracking of the amount of BC and by the fact that the questionnaire instructions were modified to reflect emotions of the last 3 days. Nevertheless, to control for avoidance and reliability, we suggest the usage of ecological momentary assessment or E-mail/text message prompting [e.g., (79)], which monitors omissions, allows for time-based and event-based sampling plans, and enables compliance to be checked in the moment. In a potential study design with ecological momentary assessment or smartphone-based prompting, participants could therefore still check their body in an typical and in an increased manner, but instead of only covering affect and body dissatisfaction in a trait-like concept every 3 days, participants might provide a state-like response directly after performing BC. This might be useful to learn more about the time course of BC. Likewise, we recommend replications with psychophysiological assessments (80), eye-tracking [cf. (81)], and interview-based data (35).

Second, future studies should conduct adapted replications in diverse subsamples. Heterogeneity in our sample was lacking in terms of gender, age, and mental health status, as we only included non-clinical women who were mostly in their early twenties and in the normal weight range. Regarding gender, similar affective responses to BC have been previously demonstrated in males in the short term (65), but the transferability to longer-term consequences has not yet been examined. Furthermore, as shown in previous research, BC is even more evident in samples with body image disturbances (15). Therefore, the inclusion of an ED sample might have magnified the present results or revealed different longer-term effects. As a further limitation, we restricted BC in terms of ED pathology. However, checking behavior occurs not only in body image disturbances, but also in various psychological diagnoses such as obsessive-compulsive, illness anxiety or panic disorder [cf. (32)], the longer-term consequences of which have not yet been fully investigated.

Third, patients with EDs are often unaware of the degree to which repeated checking has increased [(68), p. 103]. It has not yet been examined whether non-clinical individuals are also usually unaware of their BC or potential fluctuations therein. Therefore, we cannot rule out that changes, especially in the typical BC condition, are caused not only by BC itself but also by participants' heightened selective attention on the subject of BC and thus also their body. In terms of the Hawthorne effect (82), this might have led to an overestimation of the frequency of BC behavior and an increase in negative valence on the employed measures. To reduce the likelihood of selective attention bias, we implemented a 1-day baseline before the experimental manipulation started, meaning that a potential peak caused by selective attention would have become visible at the baseline level, and used this as a reference for changes throughout the typical BC condition. With respect to the small effect sizes of all dependent variables, it is further possible that the effects may have been caused by usual test inconsistencies rather than by the manipulation. Therefore, we chose instruments with a high internal consistency and test-retest reliability and adapted the time frame to reflect only about the last 3 days, corresponding to the time frame of the respective checking episode. Nevertheless, due to these limitations, our findings should be interpreted with caution, and need to be replicated in investigations employing longer BC episodes.

Fourth, regarding subsiding effects, the time course of functions of BC is vague, and research has not yet revealed when, exactly, BC leads to the proposed relief from negative affect in the short term and when it turns into the longer-term consequence of maintaining negative affect. Therefore, validation is needed to confirm the time frame within which longer-term consequences are attributable to multiple BC episodes. Additionally, a follow-up episode examining not only longer-term, but also long-term consequences should be considered in future research. Furthermore, we did not track the length of each individual BC episode. While some studies argue that BC is generally quite brief [i.e., < 2 min (64)], other authors assume it to be time-intensive [e.g., in BDD (83)]. Therefore, the length of an episode might be another potential influencing factor that needs to be examined prospectively.

Despite these limitations, the present study was the first to examine longer-term consequences of increased BC in non-clinical women. With respect to the postulates of models on the development and maintenance of EDs (24, 29), the present results suggest that the theories might be refined to include a detailed differentiation between concrete time periods that comprise short-term, longer-term and long-term consequences of BC. Furthermore, a specification of the potentially varying effect of a different frequency of BC is warranted, as we found a rather positive impact of typical BC but a negative impact of increased BC. Additionally, based on the present results, theories should consider adaptations to non-clinical populations, who – like clinical samples – tend to perform BC, but who possibly experience a more positive longer-term outcome after BC in terms of the motivation for healthy living instead of the motivation to continue with compensatory behaviors (e.g., dietary restriction).

Based on the present finding that BC leads to the maintenance of negative affect in the longer term, implications for therapy and preventive programs might be derived from our study. For patients with ED, the influence of BC on mood, which was even demonstrated in our non-clinical sample, suggests that treatment should encompass psychoeducation on the vicious cycle of BC [(68), p. 104]. Nevertheless, our findings suggest the benefit of a “just right” amount of BC. If our findings in non-clinical women are replicated and transferred to a clinical sample, the performance of BC should no longer be stigmatized as generally perilous [(68), p. 108], and its strict reduction should therefore not be aimed for in therapy. Instead, a balanced pattern of BC is potentially more helpful and protective. With respect to non-clinical or subclinical persons, low-threshold programs that detect a dysfunctional high rate of BC as an early warning sign for the development an ED may contribute to ED prevention.

## Conclusion

This study was first to examine longer-term consequences of increased BC. It supports the findings of the one previous study on the long-term impact on BC (23) by revealing that the empirical transfer of etiological ED models on BC remains limited. We found that although the longer-term increase of negative affect due to BC was in line with the assumptions of ED models, eating pathology did not change in accordance with BC. Furthermore, contrary to the assumptions on BC proposed in ED models (24), BC appeared to have an anxiolytic effect in the longer term, and typical BC even had a positive impact on general pathology. Thus, the present findings suggest a healthy impact of BC in non-clinical persons. Increased BC, however, as is more pronounced in patients with EDs (15), led to higher anxiety and higher general pathology in the longer term, which indicates negative consequences of too much BC. As BC fulfills similar functions in ED patients and non-clinical individuals, the results hint at the role of quantity as a determining factor leading to (dys)functional BC. Derived from the present results, we propose adaptations of the ED theory for non-clinical samples, insofar as a balanced BC behavior might serve as a protective factor in non-clinical individuals and could be a treatment target in patients with body image disturbances (84).

## Data Availability Statement

The raw data supporting the conclusions of this article will be made available by the authors, without undue reservation.

## Ethics Statement

The studies involving human participants were reviewed and approved by Osnabrueck University. The patients/participants provided their written informed consent to participate in this study.

## Author Contributions

AH and SV contributed to the conception and design of the study, which was elaborated with the assistance of VO and M-BV. VO wrote the first draft of the manuscript and collected and analyzed the data. All authors contributed to the revision of the manuscript and read and approved the submitted version.

## Conflict of Interest

The authors declare that the research was conducted in the absence of any commercial or financial relationships that could be construed as a potential conflict of interest.

## Publisher's Note

All claims expressed in this article are solely those of the authors and do not necessarily represent those of their affiliated organizations, or those of the publisher, the editors and the reviewers. Any product that may be evaluated in this article, or claim that may be made by its manufacturer, is not guaranteed or endorsed by the publisher.
